# Bone Marrow-Derived Mesenchymal Stem Cell Potential Regression of Dysplasia Associating Experimental Liver Fibrosis in Albino Rats

**DOI:** 10.1155/2019/5376165

**Published:** 2019-11-06

**Authors:** Yasmine H. Khalifa, Ghada M. Mourad, Wahid M. Stephanos, Sahar A. Omar, Radwa A. Mehanna

**Affiliations:** ^1^Histology and Cell Biology Department, Faculty of Medicine, Alexandria University, Dr. Fahmi Abdel Meguid Street, Mowassat Building, El Shatby, Alexandria 21561, Egypt; ^2^Center of Excellence for Research in Regenerative Medicine and Applications (CERRMA), Faculty of Medicine, Alexandria University, Alexandria 21514, Egypt; ^3^Medical Physiology Department, Faculty of Medicine, Alexandria University, Dr. Fahmi Abdel Meguid Street, Mowassat Building, El Shatby, Alexandria 21561, Egypt

## Abstract

**Objectives:**

Assessing the therapeutic efficacy of superparamagnetic iron oxide nanoparticles (SPIO) labeled bone marrow-derived mesenchymal stem cells (BM-MSCs) on experimental liver fibrosis and associated dysplasia.

**Materials and Methods:**

MSCs were obtained from 10 male Sprague-Dawley rats while 50 female rats were divided into control (CG), liver fibrosis (CCL4, intraperitoneal injection of CCl_4_ for 8 weeks), and CCL_4_ rats treated with SPIO-labeled MSCs (MSCs/CCl_4_) with and without continuing CCL_4_ injection for another 8 weeks. Assessment included liver histopathology, liver function tests, transmission electron microscopic tracing for homing of SPIO-MSCs, immunofluorescence histochemistry for fibrosis and dysplasia markers (transforming growth factor-beta (TGF-*β*1), proliferation nuclear antigen (PCNA), glypican 3)), and quantitative gene expression analysis for matrix metalloproteinase-1 (MMP-1) and tissue inhibitor of metalloproteinase-1 (TIMP-1).

**Results:**

SPIO-labeled MSCs were engrafted in the fibrotic liver and the BM/MSCs demonstrated regression for fibrous tissue deposition and inhibition progression of dysplastic changes in the liver of CCl_4_-treated rats on both the histological and molecular levels.

**Conclusion:**

BM-MSCs possess regenerative and antidysplastic potentials.

## 1. Introduction

Chronic liver disease has become an increasing health burden worldwide [1]. Liver fibrosis, following injury, is a reversible wound-healing response characterized by accumulation of extracellular matrix (ECM). The consequence of progressive fibrosis is cirrhosis, which often leads to the development of hepatocellular carcinoma (HCC) and end-stage liver failure within a period of 20 to 40 years [2].

Liver transplantation was estimated as the most effective treatment for end-stage liver fibrosis. However, transplantation is limited by shortage of organ donors and immunological rejection. MSC therapy emerged as an alternate therapeutic strategy for hepatic diseases. The beneficial effects of MSCs are attributed to the integration of multiple factors directed towards reducing excessive tissue damage in favor of repair enhancement. These factors are assumed to reduce hepatocyte apoptosis, increase regeneration, regress liver fibrosis, and enhance liver functionality [3]. Yet, several issues remain unclear: first, whether the MSCs potentials continue with persistence of the causative agent; second, the potentials of MSCs on early dysplastic changes associating liver fibrosis; and third, the issue for homing of transplanted MSCs. Besides, the molecular mechanisms of MSC trafficking to sites of injury are not fully understood which comprises one of the obstacles limiting clinical translation of MSC-based therapies. Hence, new technologies to track the behavior of MSCs are currently a persisting research priority that would facilitate the benefit from MSC-based therapy [4].

The aim of this work was to assess the efficacy of SPIO-labeled BM-MSCs in promoting regression of experimentally induced CCl_4_ liver fibrosis as well as in regressing the progression of the associated early liver dysplasia.

## 2. Materials and Methods

The present study was conducted at the Center of Excellence for Research in Regenerative Medicine Applications at Alexandria Faculty of Medicine (AFM) (CERRMA; funded by STDF/Ministry of Higher Education and Research/Egypt). The experimental animals were housed at the Animal House of the Physiology Department, AFM with food/water ad libitum. The experimental procedures followed the code of research ethics approved by the Research Ethics Committee, AFM (IRB code 0007555-FWA: No. 00018600; membership through Alexandria University in International Council of Laboratory Animal Science organization ICLAS-http://iclas.org/).

### 2.1. Isolation and Morphological Characterization of BM-MSCs

Ten male Sprague-Dawley albino rats (3 weeks old; 25–35 g) were sacrificed for isolation of femur bone marrow-derived MSCs. Using sterile syringes, bones were flushed with 5 ml complete culture medium (CCM) (low glucose Dulbecco's modified Eagle's medium (LG-DMEM) (1.0 g/L glucose; Lonza) supplemented with 10% fetal bovine serum (FBS, HyClone), 2 mM L-glutamine, and 1% penicillin/streptomycin (P/S, 10.000IU/ml/10.000 *μ*g/ml, Lonza). After passage over 70 *μ*m cell strainer, the flush was centrifuged (1200 rpm for 5 min). Cell pellet was resuspended, cultured in T-25 flask with CCM, and incubated in 5% CO_2_ at 37°C. After 48 hours, the nonadherent cells were washed by phosphate-buffered saline (PBS) and the CCM was changed once every 2–3 days. 10 days later, the primary cell culture was passaged in ratio 1 : 3 to reach 80%–90% confluence using 0.25% trypsin-EDTA solution (170.000 U/l-200 mg/l, Lonza). MSCs at passage 3 (P3) were resuspended at 1 × 10^6^ cells/ml in CCM for transplantation [5].

### 2.2. Colony Forming Unit Fibroblast Assay (CFU)

100 cells were plated/incubated on a six-well plate in CCM. After 14 days, the medium was discarded, and the cells were washed with PBS and fixed and stained using Crystal Violet (Sigma-Aldrich, USA) at 3% (w/v). The number of visible colonies per well was counted. Colonies displaying five or more cells were scored under the inverted microscope taking in consideration the optimal CFU potential range (over 40%). The “CFU potential” was calculated as equal to the number of colonies formed/number of cells plated ×100 [6].

### 2.3. Characterization of BM-MSCs by Fluorescence-Activated Cell Sorting (FACS)

At P3, MSCs were characterized by using fluorescent-labeled monoclonal antibodies (mAb) for CD90, CD44, and CD34 surface markers. Adherent cells were trypsinized, washed with PBS, and incubated, at room temperature for 30 min in dark, with monoclonal allophycocyanin-conjugated antibody for CD90 (Anti-Thy1.1) (Abcam, Cambridge, UK), monoclonal phycoerythrin- (PE-) conjugated antibody for CD44 (Abcam, Cambridge, UK), and monoclonal PE-conjugated antibody for CD34 (Abcam, Cambridge, UK). Subsequently, cells were washed thrice with PBS and resuspended in 500 *μ*l FACS buffer. Immunofluorescence on the viable cells was performed using BD FACS Calibur flow cytometer equipped with Cell Quest software (Becton Dickinson, New Jersey, USA) [7].

### 2.4. In Vitro BM-MSCs Labeling by Nanoparticles for Tracking

Cultured BM-MSCs were labeled with SPIO (dextran-coated ferumoxide (Fe_3_O_4_); FeraTrack Direct Contrast Particles, Miltenyi Biotec, Germany) and further incubated in 5% CO_2_ at 37°C. SPIO mean particle size was 150 nm in a dose 100 *μ*g Fe/1×10^6^ cells. Cell viability of labeled BM-MSCs was examined by dye exclusion test (0.04% trypan blue solution) [8]. Before SPIO labeling, 0.5–1×10^6^ cells/9.8 cm^2^ of P3 cells in a 6-well plate were cultured (2 ml medium one day before labeling to reach 90% confluent). CCM was exchanged with serum-free media, followed by adding 40 *μ*l of the FeraTrack particles dropwise, and incubated in 5% CO_2_ at 37°C. After 24 h, cells were washed twice with PBS and CCM was added [9].

### 2.5. Prussian Blue Staining of the Labeled BM-MSCs

The labeled BM-MSCs were washed twice with PBS fixed in 4% formaldehyde for 10 min, rewashed twice in PBS, stained by Prussian blue (20 min), and then washed three times in distilled water. Counter staining by eosin (30 sec) was followed by double wash, dehydration, clearing, and mounting with cover slip [10].

### 2.6. CCl_4_-Induced Liver Fibrosis Rat Model

Fifty adult female Sprague-Dawley albino rats (6–8 weeks, 150–200 g) were divided into the following groups.

#### 2.6.1. Control Groups (CG)

Control groups (CG) (*n* = 20 rats) were divided into four subgroups. CG1 (*n* = 5) rats received single portal vein infusion (PVI) of 1 ml PBS (vehicle of SPIO) slowly over 5 min. CG2 (*n* = 5) rats were given an intraperitoneal (IP) injection of olive oil (vehicle for CCL_4_) as 1 ml/kg body weight twice/week for 8 successive weeks. CG3 (*n* = 5) rats received single PVI of 4 *μ*l SPIO/1 ml PBS by slow infusion over 5 min. CG4 (*n* = 5) rats received single PVI of 1 ml CCM (vehicle of BM-MSCs) slowly over 5 min. Rats of the four control subgroups were sacrificed after 8 weeks [8, 11].

#### 2.6.2. Experimental Group (EG)

Experimental group (*n* = 30 rats) were divided into three subgroups. (1) Liver fibrosis group (*n* = 10 rats) rats were injected intraperitoneally with CCl_4_ (99.5% purity, Chema tech, Tianjin, China) diluted 1 : 1 in olive oil at a dose of 1 ml/kg body weight twice/week for 8 successive weeks [12]. Rats that died during induction were substituted. (2) MSCs treated with maintained CCl_4_ exposure group (MSCs + CCl_4_G) (*n* = 10 rats) were treated with single PVI of SPIO-labeled BM-MSCs. 1 × 10^6^ labeled BM-MSCs/rat in 1 ml of CCM were infused simultaneously with CCl_4_ administration to assess the effect of SPIO-BM-MSCs on progression of liver fibrosis with the persistence of the cause (CCl_4_). (3) MSCs-treated group (MSCsG) (*n* = 10 rats) rats received single PVI of SPIO-labeled BM-MSCs after stopping CCl_4_, 24 hours after the last CCl_4_ injection. The rats were sacrificed after 8 weeks [13].

### 2.7. Technique for PVI and Cell Transplantation

Upon rat anesthesia with ketamine hydrochloride 100 mg/kg IM/Xylaject 5 mg/kg (Trittau/Germany and Pharmaceutical Company/Egypt, respectively), a midline abdominal incision was performed under sterile surgical procedures to expose the portal vein. The tested material was slowly infused by insulin syringe needle into the portal vein over 5 minutes [12].

### 2.8. Biochemical Analysis of Liver Function Tests

Before euthanizing rats, blood samples were collected from the inferior vena cava in plain tube vacutainers and left for 30 min to clot. Serum was isolated within one hour by centrifugation at 2000 g for 10 min. Sera from all groups were stored at −80°C and then an automated chemical analyzer (7600; Hitachi, Tokyo, Japan) was used according to the manufacturers' instructions [14].

### 2.9. Real-Time Quantitative Polymerase Chain Reaction (qPCR) for Gene Expression Levels of MMP-1 and TIMP-1

Homogenized liver tissues (Glas-Col homogenizer, USA) from control and experimental groups were subject to RNA extraction using commercially available kit (Thermo Scientific Gene JET RNA Purification Kit). Rat primers for MMP-1, TIMP-1, and *β*-actin (Sigma Aldrich, California, USA) were used with reference to manufacturer's datasheet. qPCR for *β*-actin was the positive control of DNA quality. qPCR amplification, data acquisition, and analysis were carried out using the real-time detection system and software (Applied Biosystems 7500) [15].

### 2.10. Histopathological Assessment by Light Microscopy

#### 2.10.1. Microtechniques.  

5–6 *μ*m thick paraffin sections from liver of all rat groups were assessed by light microscopy (BX41, Olympus) using hematoxylin and eosin stain (H&E), Prussian blue for the detection of SPIO, and Gomori's trichrome for the deposition of collagen [16].

#### 2.10.2. Immunohistochemical Study

Paraffin sections were stained with sheep polyclonal IgG anti-human glypican 3 (AF2119, R&D Systems, Minneapolis, Minnesota, USA) by routine immunohistochemical methods using DAB chromogen (diaminobenzidine) and hematoxylin counterstain [17].

#### 2.10.3. Immunofluorescence Assay

*(1) Transforming Growth Factor TGF-β1 Monoclonal Anti-Mouse IgG1 Primary Antibody (R&D Systems, Minneapolis, Minnesota)*. The primary antibody was incubated with paraffin liver sections and mounted on positively charged glass slides. It was conjugated to the goat anti-mouse secondary antibody Alex Fluor 555(Invitrogen, Cat # A-21422; Excitation/emission wave lengths 555/580 nm) using confocal laser scanning microscopy (Leica TSC SPE II/DMi 8). Hoechst 33342 was used as fluorescent nuclear stain (Thermo Fisher Scientific, excitation/emission wave lengths 361/497 nm) [18].

*(2) Liver Sections Were Examined for Antiproliferating Cell Nuclear Antigen PCNA Primary Mouse Monoclonal Antibody (Sigma Aldrich, Saint Louis, USA)*. The anti-PCNA was incubated with paraffin liver sections, mounted on positively charged glass slides and conjugated with goat anti-mouse secondary antibody Alex Fluor 555 and Hoechst 33342 as mentioned in the previous paragraph [19].

### 2.11. Morphometric Study

Using NIH Fiji©program (version 1.51k, Wayne Rasband, National Institutes of Health, Maryland, USA), the area percentage of collagen density in Gomori's trichrome-stained sections, the number of positive cells in sections stained with anti-glypican 3 immunohistochemistry, and the cell fluorescence intensity in sections immune-stained with TGF*β* and PCNA were measured in random five fields/section in five sections in six rats from each group at magnifications 200, 400, and 63, respectively [20].

### 2.12. Transmission Electron Microscopy

TEM for tracing transplanted SPIO-labeled BM/MSCs: 1 mm^3^ liver specimens from liver of groups receiving SPIO-labeled BM/MSCs were immediately fixed in 1% glutaraldehyde for 2 hours at room temperature followed by 4% formaldehyde-1% glutaraldehyde fixative for 48 hours. The specimens were processed, mounted on meshed copper grids, and stained by lead citrate/uranyl acetate to be examined by Joel TEM, Electron Microscopy Unit, Faculty of Science, Alexandria University [21].

### 2.13. Statistical Analysis

The biochemical, PCR, and morphometric results were analyzed using IBM SPSS software package version 20.0 (IBM Corp., Armonk, NY). They were expressed as mean ± standard deviation (SD). Significant differences were determined using ANOVA and were considered significant when *p* ≤ 0.05 [22].

## 3. Results

### 3.1. Morphological Characterization of BM-MSCs

**Figure 1:**
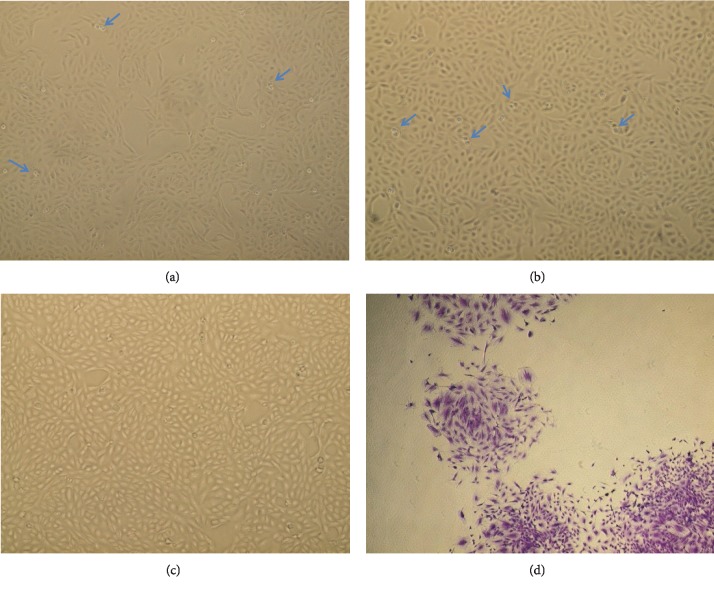
(a–c) Morphological characterization of BM-MSCs. Spindle-shaped, fibroblast-like cells. (a) P1 showing the adherent spindle-shaped MSCs cells 60% confluent (100×). (b) P2 showing the spindle-shaped MSCs with 70% confluent (100×). (c) P3 showing the spindle-shaped MSCs with 90% confluent (100×). (d) CFU assay, P3 Crystal Violet stain showing 4 colonies (100×). Blue arrows show rounded cells undergoing mitotic division (proliferating cells).

24 hrs after culturing, most of the BM-MSCs were rounded and adhered to the culture flask surface within 72 hrs; then a monolayer of spindle-shaped cells is displayed and showed 80–90% confluence by the 8^th^–10^th^ day (Figures 1(a)–1(c)).

### 3.2. Colony Forming Unit Fibroblast Assay

CFU-F assay provided evidence of MSCs proliferation with clonogenic capacity of 83% ± 2.32 colonies/100 BM-MSCs within 14 days of culture (Figure 1(d)).

### 3.3. Immunophenotyping of P3 BM-MSCs

**Figure 2:**
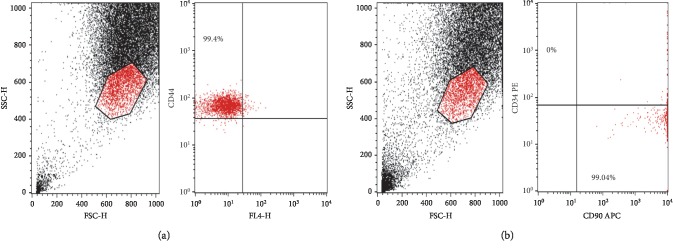
(a, b) Flow cytometric analysis of cell-surface markers of BM-MSCs at passage 3. (a) 99.4% of the cultured cells expressed the mesenchymal cell marker CD44 in upper left quadrant. (b) 99.04% of the cultured cells expressed the mesenchymal cell marker CD90 in lower right quadrant, while they were negative for the CD34 hematopoietic marker, upper left quadrant.

Immunophenotyping of P3 BM-MSCs showed that 99.4% and 99.04% of the cultured cells expressed the mesenchymal CD44 and CD90 markers, respectively, but were negative for the CD34 hematopoietic marker (Figures 2(a) and 2(b)).

### 3.4. Efficiency of BM-MSCs Labeling with SPIO

**Figure 3:**
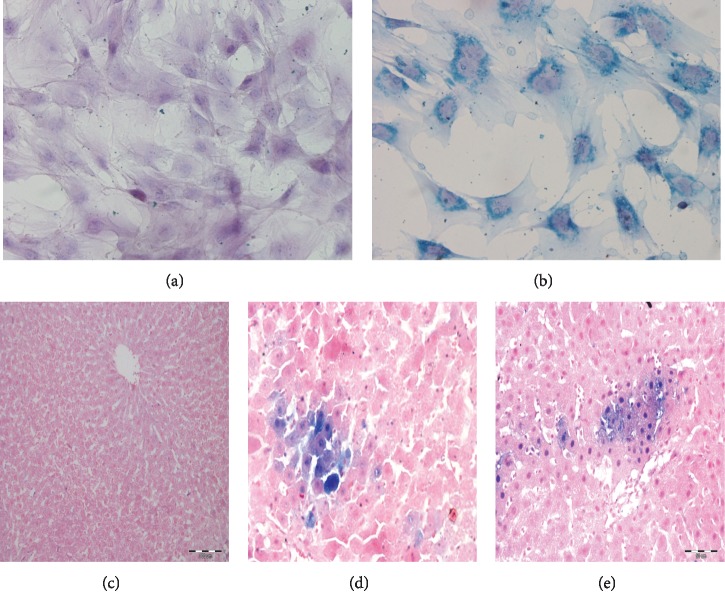
(a) Unlabeled control BM-MSCs (Prussian blue, 400×). (b) SPIO-labeled BM-MSCs showing dark blue aggregates of the iron nanoparticles in the cytoplasm (Prussian blue, 400×). (c–e) Light micrographs of liver tissue stained by Prussian blue. (c) LFG showing negative reaction (200×). (d, e) MSCs + CCl_4_G and MSCsG showing blue deposits in the cytoplasm of groups of cells among the hepatocytes (mic. mag.: (a) ×200; (b, c) 400×).

The uptake of SPIO nanoparticles was depicted in Prussian blue stained liver sections as aggregates of cells with bluish deposits in the cytoplasm of transplanted MSCs scattered among the unstained hepatocytes (Figures 3(a) and 3(b)).

### 3.5. Biochemical Study of Liver Enzymes, Total Bilirubin, and Serum Albumin

After 8 weeks of CCl_4_ treatment, ALT and AST enzymes were significantly increased in the LFG compared to control rats (*p* ≤ 0.01). BM-MSC transplantation significantly reduced the serum levels of ALT and AST in sera of rats from MSCsG compared to LFG and MSCs + CCl_4_G (*p* ≤ 0.01) but showed no significant difference to the control rats. For the MSCs + CCl_4_G, a significant increase in the levels of ALT and AST persisted in comparison to control group but the levels of ALT and AST were significantly lower compared to the LFG (*p* ≤ 0.01). There was also a significant decrease in mean value of serum albumin levels in LFG and MSCs + CCl_4_G (*p* ≤ 0.01), which was recovered to control levels in the MSCsG (after withdrawal of CCl_4_). Total bilirubin level was significantly increased in LFG and MSCs + CCl_4_G in comparison to control group but was significantly reduced in the MSCsG approaching the level in the control group (*p* ≤ 0.01; Table 1).

### 3.6. Results of qPCR

The mean values for gene expression were reduced for MMP-1 and increased for TIMP-1 in LFG compared to control group. Similar results occurred for the MSCs + CCl_4_ group. On the other hand, values for MSCs + CCl_4_G were significantly increased for MMP-1 and decreased for TIMP-1 in comparison to LFG (*p* ≤ 0.01). On withdrawing CCl_4_, the MSCsG recovered a significant increase in the expression of MMP-1 with concomitant decrease in TIMP-1 compared to LFG and MSCs + CCl_4_G (*p* ≤ 0.01) but with no significant difference to the CG (Table 2).

### 3.7. Histological and Morphometric Results of Liver Fibrosis

CCl_4_ toxicity caused 20% mortality rate of the rats after eight weeks of administration.

#### 3.7.1. Histological Examination of H&E Stained Liver Sections

**Figure 4:**
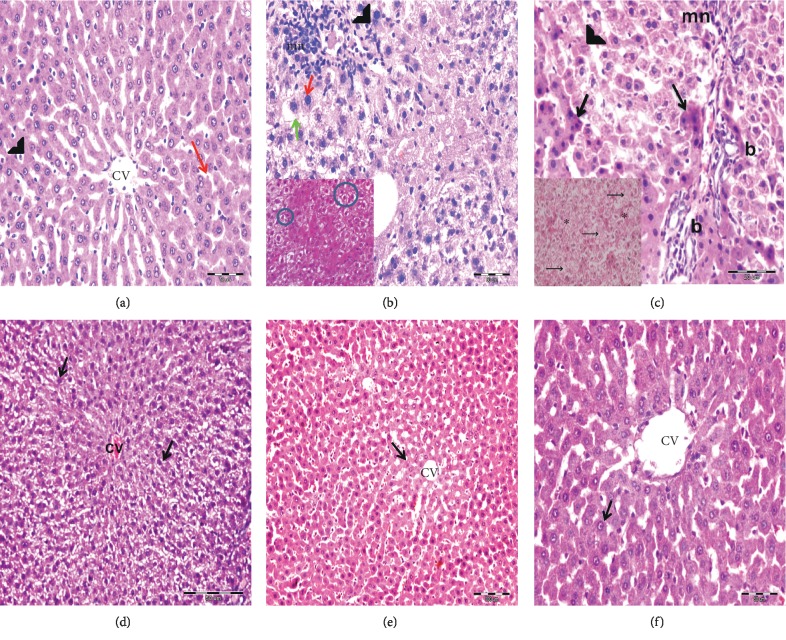
Light photomicrographs stained by H&E. (a) CG showing the cords of hepatocytes radiating from the central vein (CV) and separated by slit-like sinusoidal spaces (red arrow) lined by endothelial cells (arrow head) (mic. mag.: 400×). (b, c) LFG showing the disorganized hepatic architecture. Many centrilobular hepatocytes have swollen vacuolated cytoplasm (green arrow) with obliterated blood sinusoids in between. Periportal hepatocytes with vacuolated cytoplasm and karyolitic nuclei (arrow head) are observed. Many hepatocytes have deeply stained eosinophilic cytoplasm and dark nuclei (black arrow). Proliferated bile ducts (b) and periportal mononuclear cellular infiltration (mn) are also seen. Few hepatocytes show increased nucleocytoplasmic ratio (red arrow) (mic. mag.: 400×). Inset in (b) shows rosette shaped aggregates (circle) (mic. mag.: 400×). Inset in (c) shows cells having vacuolated cytoplasm and karyolitic nuclei (arrows) with obliterated blood sinusoids in between (^*∗*^) (mic. mag.: 400×). (d) MSCs + CCl_4_G showing disorganized hepatic architecture with obliterated sinusoids. Many hepatocytes reveal swollen vacuolated cytoplasm and deeply stained nuclei (arrow) radiating from congested central vein (CV) (mic. mag.: 200×). (e, f) MSCsG showing restored architecture of the hepatic lobules. The hepatocytes are arranged in cords radiating from the central vein (CV) and separated by patent blood sinusoids. Centrilobular hepatocytes appear with eosinophilic granular cytoplasm and rounded vesicular nuclei (arrow) ((e): 200×; (f): 400×).

Histological examination of H&E stained liver sections of control subgroups revealed normal hepatic architecture with average stroma and structural components in the portal tracts (Figure 4(a)). Similar unaltered histological structure was also observed in control rats receiving PBS and SPIO. Following CCl_4_ treatment, the LFG exhibited disorganized hepatic architecture with hepatocytes showing variable degrees of centrilobular degenerative changes involving wide spread segments of several hepatic lobules and individual apoptotic cells. There were foci of hepatocytes exhibiting dysplastic changes in the form of increased nucleocytoplasmic ratio and peculiar arrangement in acinar-like structures acquiring rosette shaped configurations. Vascular congestion, cellular infiltrates, and proliferation of bile cholangioles were concomitant observations in the CCl_4_-treated liver sections (Figures 4(b) and 4(c)).

On transplantation of BM-MSCs with continuation of CCl_4_ treatment (MSCs + CCl_4_ G), the liver sections showed persistence of the CCl_4_ hepatic lesions (Figure 4(d)), whereas in rats with BM-MSCs and withdrawal of CCl_4_ (MSCsG) the architecture of hepatic lobules and that of most of the hepatocytes were restored (Figures 4(e) and 4(f)).

#### 3.7.2. The Gomori's Trichrome Stained Sections

**Figure 5:**
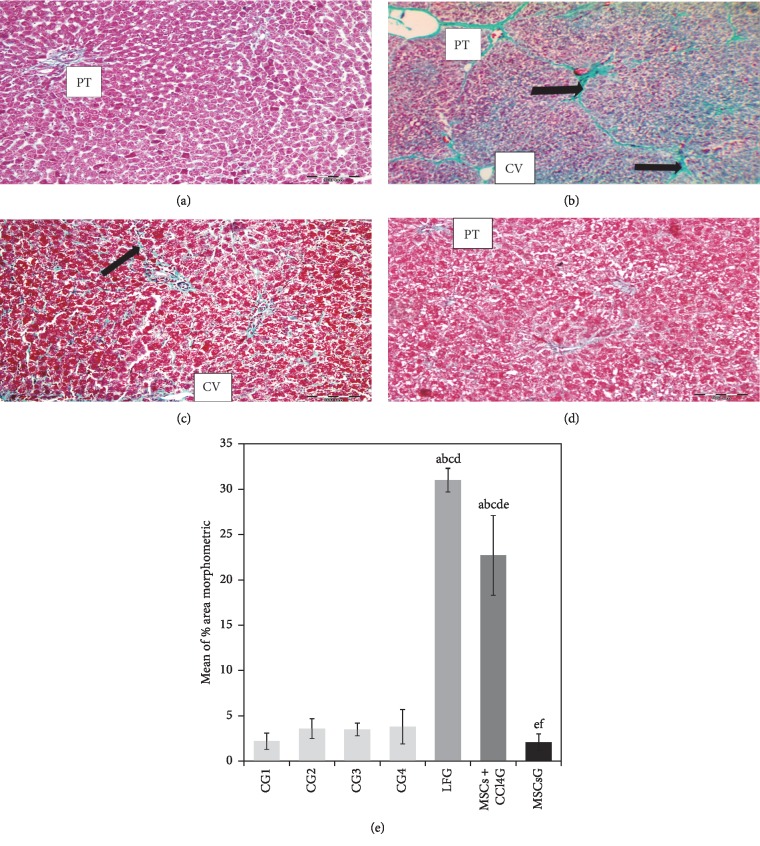
(a–d) Light photomicrographs of rat liver sections stained by Gomori's trichrome. (a) CG showing the average green fibers in the stroma of the portal tract (PT). No fibers are seen between hepatocytes. (b) LFG showing the prominent deposits of collagen fibers around the central veins (CV), in the portal tract (PT) and between hepatocytes (arrow). (c) MSCs + CCl_4_G showing the fine collagen fiber deposits around the central veins (CV) and between the hepatic cords (arrow). (d) MSCsG showing the minimal deposits of collagen fibers in the portal tracts (PT) of hepatic lobules (mic. mag.: 200×). (e) A morphometric study for the area percentage of collagen. Values represent mean ± SD. Different letters indicate significant statistical differences at *p* ≤ 0.05. ^a^Significant compared with CG1. ^b^Significant compared with CG2. ^c^Significant compared with CG3. ^d^Significant compared with CG4. ^e^Significant compared with LFG. ^f^Significant compared with MSCs + CCl_4_G. It revealed that CCl_4_ treatment led to significant increase in mean area percentage of collagen which was reduced following the BM-MSCs treatment.

The Gomori's trichrome stained sections of the LFG demonstrated deposition of green stromal fibers around the central veins, in the portal tracts and between the hepatocytes compared to the control liver sections (Figures 5(a) and 5(b)). In the MSCs + CCl_4_G, the fibrous deposition was less intense compared to the LFG (Figure 5(c)). Eight weeks after BM-MSCs treatment (MSCsG), only minimal fibrous tissue was detectable apparently similar to the control group. The results were statistically confirmed by values obtained from morphometric analysis (Figure 5(d)).

#### 3.7.3. Tracking of Transplanted MSCs for Engraftment in the Liver

Tracking of transplanted MSCs for engraftment in the liver was done for rats of MSCs + CCl_4_G and MSCsG, at 60 days of the transplant by TEM (Figures 3(c)–3(e)).

**Figure 6:**
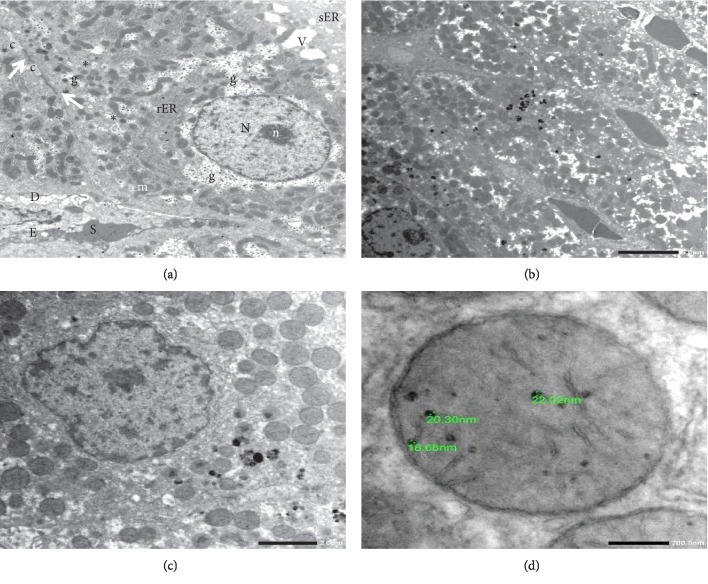
Transmission electron photomicrograph. (a) Normal control hepatocyte demonstrating average cytoplasmic structures. N = nucleus; n = nucleolus; m = mitochondria; g = glycogen rosettes; rER = rough endoplasmic reticulum; sER = smooth endoplasmic reticulum; v = vacuole. (b) MSCs loaded with electron dense SPIO particles (arrow) inserted between the hepatocytes and adjacent to the liver sinusoids. (c) Higher magnification of MSCs loaded with SPIO particles. (d) High power of mitochondria in MSCs revealing SPIO dense particles of different sizes in its matrix (lead citrate–uranyl acetate stain; mic. mag.: (a) 5000×, (b) 2000×, (c) 3000×, and (d) 10000×).

The SPIO-loaded BM/MSCs were recognized by lacking the cytoplasmic components characteristic for hepatocytes as glycogen granules, rough endoplasmic reticulum, and smooth endoplasmic reticulum. The MSCs were usually located close to the hepatic sinusoids among the adjacent hepatocytes. The cytoplasm of MSCs demonstrated the engulfed SPIO particles as rounded electron dense particles of variable sizes. The particles were also depicted inside the mitochondria of the MSCs. On the contrary, hepatocytes did not demonstrate SPIO particles in their cytoplasm (Figures 6(a)–6(d)).

#### 3.7.4. Immunohistochemical Results for Glypican 3 Activity

**Figure 7:**
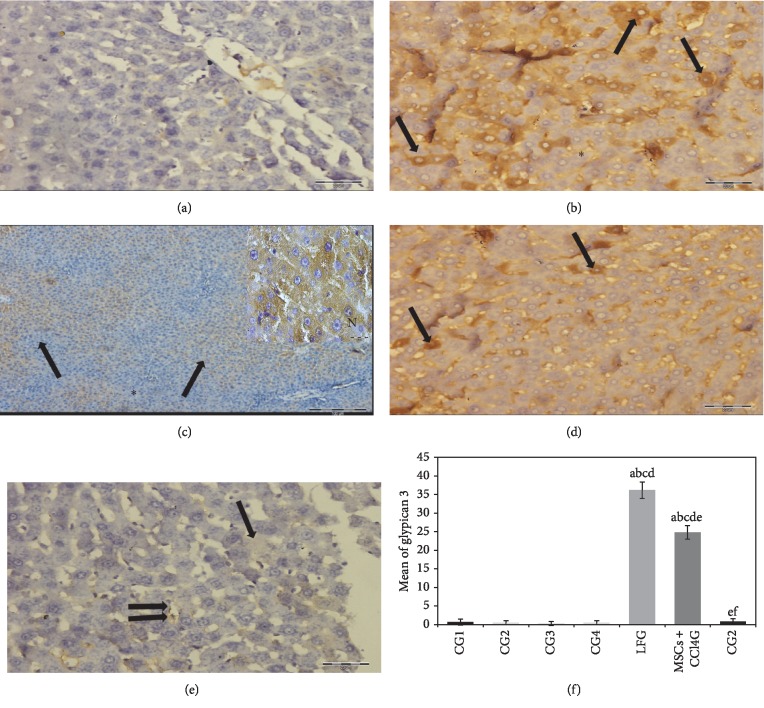
(a–e) Rat liver stained with anti-glypican 3 primary antibody. (a) CG demonstrating negative reaction (mic. mag.: 400×). (b, c) LFG shows positive brownish reaction scattered in many foci of hepatic lobules (arrows) while other areas show negative reaction (black asterisk) (mic. mag.: (b) 400×; (c) 100×). Inset in (c) is an oil emersion high power of hepatocytes depicting the cytoplasmic brown granular deposits of glypican 3 (white asterisk). Note that the nuclei of hepatocytes are devoid of brown deposits (N) (mic. mag.: 1000×). (d) MSCs transplanted into rat liver with continuous CCl_4_ exposure (MSCs + CCl_4_) demonstrating evident reduction in hepatocytes with positive brown cytoplasmic deposits (arrows) compared to (b) (mic. mag.: 400×). (e) MSCs transplanted into rat liver after cessation of CCl_4_ exposure (MSCsG) depicting faint positive reaction in individual hepatocyte (arrow) and in the lining of hepatic sinusoids (double arrow) (mic. mag.: 400×) (immunohistochemistry anti-glypican 3 antibody, DAB chromogen, and hematoxylin counterstain). (e) A morphometric study for the number of positive cells for anti-human glypican 3. Values represent mean ± SD. Different letters indicate significant statistical differences at (*p* ≤ 0.05). ^a^Significant compared with CG1. ^b^Significant compared with CG2. ^c^Significant compared with CG3. ^d^Significant compared with CG4. ^e^Significant compared with LFG. ^f^Significant compared with MSCs + CCl_4_G. It revealed that CCl_4_ treatment led to significant increase in the number of positive cells which was reduced following BM-MSCs treatment.

Statistical analysis of mean values for hepatocytes with positive glypican 3 reaction illustrated highest reaction intensity in hepatocyte cytoplasm of rat liver from LFG and MSCs + CCl_4_G indicative for the inset of dysplastic changes in comparison to the MSCsG and CG (Figures 7(a)–7(e)).

#### 3.7.5. Immunofluorescence Staining

**Figure 8:**
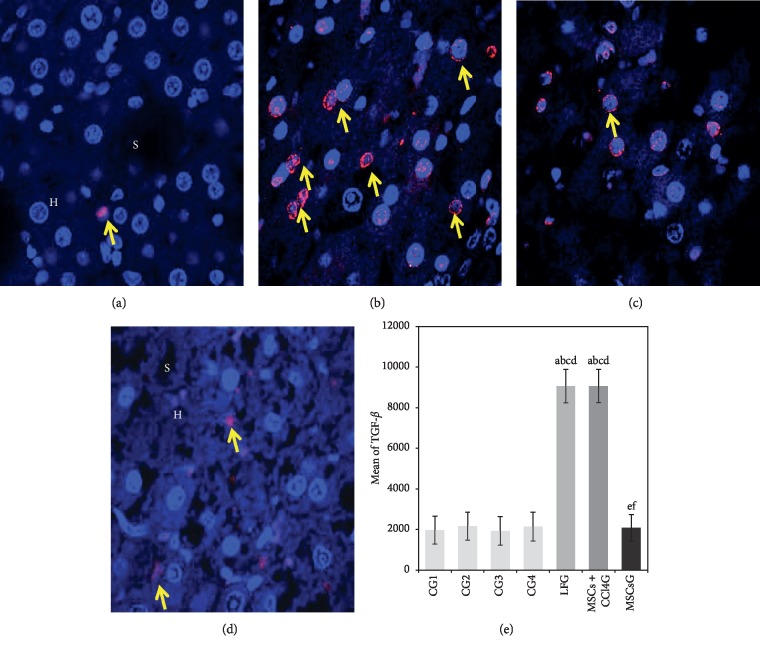
(a–d) Light photomicrographs of immunofluorescence-stained liver sections for the expression of TGF*β*1. (a) CG showing the minimal expression of red fluorescent TGF*β*1 (arrow) mainly in the cytoplasm of individual hepatocytes (H) and sinusoids (S). (b) LFG showing the strong expression of red fluorescent TGF*β*1 in the perinuclear cytoplasm of many hepatocytes (arrow). (c) MSCs + CCl_4_G showing the moderate expression of red fluorescent TGF*β*1 in the perinuclear cytoplasm of hepatocytes (arrow). (d) MSCsG showing the minimal expression of red fluorescent TGF*β* in the cytoplasm of few hepatocytes (arrow) (confocal microscopy, anti-TGF*β*1, Hoechst nuclear stain, mic. mag.: 63×). (e) A morphometric study applying manual counting for the mean number of cell fluorescence for TGF*β*1. Values represent mean ± SD. Different letters indicate significant statistical differences at *p* ≤ 0.05. ^a^Significant compared with CG1. ^b^Significant compared with CG2. ^c^Significant compared with CG3. ^d^Significant compared with CG4. ^e^Significant compared with LFG. ^f^Significant compared with MSCs + CCl_4_G. It reveals that CCl_4_ treatment leads to significant increase in the total cell fluorescence of TGF*β*1 which is reduced following BM-MSCs treatment.

*(1) TGF-β1*. Statistical analysis of positive TGF-*β*1 red fluorescent signaling in hepatocytes perinuclear cytoplasm showed highest mean values in the LFG and MSCs + CCl_4_ G in comparison to low mean values for the MSCsG, thus reflecting the variation in the profibrogenic role of TGF-*β*1 in response to CCl_4_ administration and to the effect of transplanted MSCs (Figures 8(a)–8(e)).

**Figure 9:**
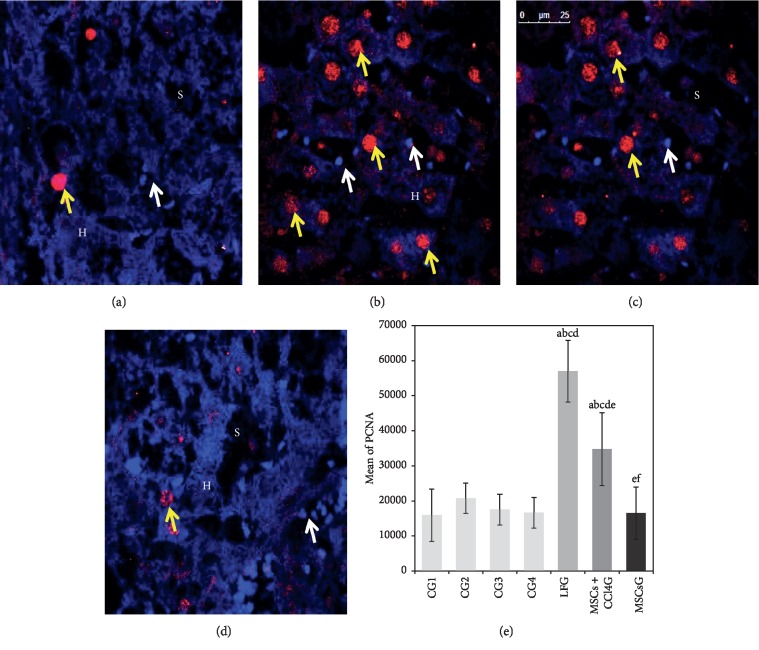
(a–d) Light photomicrographs of immunofluorescence-stained liver sections for the expression of PCNA. (a) CG showing the expression of red fluorescent PCNA in the nuclei (yellow arrow) of individual hepatocytes (H). The nuclei of cells (white arrow) lining the sinusoids (S) show Hoechst nuclear stain but no PCNA reactivity. (b) LFG showing the expression of red fluorescent PCNA in the nuclei (yellow arrow) of many hepatocytes. (c) MSCs + CCl_4_G showing the expression of red fluorescent PCNA (yellow arrow) in the nuclei of multiple hepatocytes. (d) MSCsG showing evident reduction in the expression of red fluorescent PCNA in the nuclei (yellow arrow) of hepatocytes (confocal microscopy, anti-PCNA, Hoechst nuclear stain, mic. mag.: 63×). (e) A morphometric analysis applying manual counting for the mean of hepatocyte nuclei with positive red fluorescence for PCNA. Values represent mean ± SD. Different letters indicate significant statistical differences at *p* ≤ 0.05. ^a^Significant compared with CG1. ^b^Significant compared with CG2. ^c^Significant compared with CG3. ^d^Significant compared with CG4. ^e^Significant compared with LFG. ^f^Significant compared with MSCs + CCl_4_G. It reveals that CCl_4_ treatment leads to significant increase in the total cell fluorescence for PCNA which is reduced following BM-MSCs treatment particularly in the MSCs G compared to the MSCs + CCl_4_G (with continuous exposure to CCl_4_).

*(2) PCNA*. Statistical analysis of hepatocytes showing positive nuclear PCNA fluorescent signaling illustrated highest mean values among rat liver in LFG, less evidently in the MSCs + CCl_4_G and minimal values for the MSCsG (Figures 9(a)–9(e)).

## 4. Discussion

MSCs have been directed to improve the outcome of allogenic transplantation, promoting hematopoietic engraftment [23], and to hamper graft-versus-host disease [24].

The present study was designed to validate the effect of BM-MSCs transplantation in a rat model with CCl_4_-induced liver fibrosis on morphometric and molecular basis. The implemented CCl_4_ model is well established to mimic the pathogenesis of liver fibrosis in human including inflammation, fibrogenesis, and regeneration [25]. CCl_4_ is metabolized by cytochrome P450 in the liver, with release of free radical CCl_3_ leading to hepatocyte degeneration that initiates inflammatory responses and vascular congestion in the liver [26].

Liver fibrosis, as evidenced by Gomori's trichrome-stained liver sections, can be attributed to the involvement of oxidative stress occurring as a result of an imbalance between the production and clearance of reactive oxygen species (ROS) [27]. The latter can directly affect the behavior of myofibroblasts by upregulating profibrogenic genes, including procollagen type I and TIMP1, thus contributing to excessive tissue remodeling and fibrogenesis [28].

On the molecular level, MMP-1 expression and TIMP-1 expression were assessed as indicators for extracellular matrix (ECM) remodeling. MMPs are responsible for the degradation of ECM proteins. They are secreted as inactive proenzymes and are modulated by endogenous proteinase inhibitors known as TIMPs [29]. Activated hepatic stellate cells (HSCs) are considered the sources of MMPs and TIMPs [30]. TIMP-1 has antiapoptotic effects on HSCs; thus, it induces fibrogenesis by promoting fibrogenic cell survival [31].

The CCl_4_-induced alteration in the hepatic parenchyma and stroma caused the development of centrilobular degenerative hepatocyte, particularly steatosis [32]. CCl_3_ and other CCl_4_-related ROS were proved to alter lipid metabolism in the liver. They inhibit lipid transport leading to cytoplasmic accumulation of lipids in hepatocytes smooth endoplasm, formation of lipid vacuoles, and, hence, fatty liver or steatosis. In addition, other hepatocytes showed cytoplasmic depletion and nuclear pyknosis, especially in the MSCS + CCl_4_G. Boll et al. [33] postulated that CCl_4_ injury is dose and duration dependent. Prolonged exposure to CCl_4_ leads to progression consequences of the altered lipid metabolism, mainly lipid peroxidation of cell membranes with subsequent necrosis.

Apoptosis was also frequently identified among hepatocytes of CCl_4_-treated rats. Hubscher et al. [34] explained the occurrence of apoptosis secondary to the inflammatory response triggered by liver toxicants. The latter trigger the release of cytokines by the liver macrophages leading to the initiation of the apoptosis cascades in hepatocytes, especially in steatotic hepatocytes. The hypothesis relating hepatocyte apoptosis to fibrosis suggests that apoptotic bodies released by hepatocytes apoptosis are phagocytosed by Kupffer cells, HSCs, and adjacent hepatocytes. This results in the production of chemokines and cytokines, including TGF-*β*1, which in turn activates HSCs leading to fibrosis [35]. In the present study, we demonstrated the increased expression of TGF-*β*1 in the CCl_4_-treated liver sections, which confirmed its involvement in apoptosis and the related liver fibrosis. TGF-*β*1 is a primary mediator in liver fibrosis. It promotes HSCs transition into myofibroblasts and stimulates the synthesis of collagen-1. It also inhibits ECM degradation by stimulating the production of TIMPs [36].

The CCl_4_ rat liver sections revealed also evidences for biliary duct proliferation. The finding has been related to ROS that trigger mitosis of the epithelial lining of cholangioles and their elongation, thus resulting in increased encountering of bile ductules cross sections [37]. Lopez Panqueva [38] explained that the biliary reaction represents one algorithm of liver regeneration in response to liver damage. Toxic liver injuries are associated with augmented biliary secretion as a mechanism for elimination of the detoxification metabolites. Subsequently, the proliferation and elongation of biliary ductules occur to enhance the drainage of excess bile.

The CCl_4_-induced liver insults were furthermore reflected on the altered liver function as indicated by serum ALT and AST enzymes, serum albumin, and total serum bilirubin. The raised serum levels of liver enzymes ALT and AST in CCl_4_-treated rats are explained by the hepatocyte degeneration and altered mitochondrial membrane permeability in response to lipid peroxidation by the CCl_4_-ROS, thus causing escape of the cytoplasmic enzymes into the circulation [39]. Moreover, there was an increase in the total serum bilirubin as explained above in response to the enhanced detoxification activity of hepatocytes to eliminate CCl_3_ metabolites [38]. On the other hand, a significant drop in serum albumin level was demonstrated reflecting the suppressed albumin synthesis as a result of CCl_4_-induced hepatocyte degeneration [40].

Hepatic dysplasia was evidenced as a concomitant alteration associating the CCl_4_-induced liver fibrosis even if still not reaching the extent of overt cirrhosis. The fibrosed rat livers revealed microscopic foci of hepatocytes dysplasia varying between anisonuclear changes, particularly increased nucleocytoplasmic ratio as well as rosette shaped arrangement around microvascular spaces. This was further confirmed by the significant increased expression of PCNA and glypican 3 in hepatocytes of the CCl_4_-treated rats. Bibbo and Wilbur [41] described similar dysplastic transformation in chemically induced liver insults. CCl_4_ has been suspected for its carcinogenic potentials. Schawkat and Reiner [42] postulated that liver fibrosis and steatosis are risk factors for the development of hepatocellular carcinoma (HCC).

Glypican 3 (GPC3) is a transmembrane heparan sulfate proteoglycan anchored to the cell membrane of hepatocytes in 70%–90% of all HCCs. It is normally detected in the fetal liver but absent in the healthy adult liver [43]. It plays a role in tissue-dependent regulatory cellular signaling for both stimulatory and inhibitory growth pathways. Recently, glypican 3 has been evaluated as a marker for HCC due to its overexpression in the liver during growth. Immunotherapy targeting GPC3 is currently investigating its reliability as a biomarker for the detection, prediction of prognosis, and treatment of HCC [44].

PCNA is a 36 kDa nuclear protein playing a key role in DNA damage repair, cell cycle control, cell survival, chromatin assembly, gene transcription, and sister-chromatid cohesion. PCNA levels are low in quiescent cells but are raised during DNA replication, so it is a sensitive index of proliferation. Overexpression of PCNA is usually consistent with tissues progressing from normal cells to hyperplasia, dysplasia, and premalignant and malignant lesions [19, 45].

Transplantation of rat BM-MSCs was assessed for their potentials to ameliorate the CCl_4_-induced liver injuries. Repair of the injured tissues of liver fibrosis is influenced by multiple factors including the delivery route, the sources of transplanted cells, the number of infused cells, culture conditions, gene modification of MSCs, and other potential factors [46].

Transportal infusion of the isolated BM-MScs proved to be a successful approach ensuring homing of the transplanted MSCs into the liver parenchyma. The infused SPIO-labeled MSCs were demonstrated both in Prussian blue liver sections and by TEM observation of MSCs loaded with SPIO nanoparticles among hepatocytes. Nanotechnology-based cell tracking methods provide nontoxic, noninvasive, and clinically applicable solutions for tracing of cells after injection [47]. Local infusion of MSCs through the portal vein comprises a convenient and feasible way of MSCs transplantation. Similar studies reported that local MSCs injection to the site of injury or near the site of injury provides more number of cells and increases its functional capacity [48]. Many experimental studies demonstrated that the majority of intravenously administered MSCs lodge in the lung microvasculature upon the first pass through the circulation, regardless of the presence or absence of lung-specific injury [49]. There are controversial results concerning the transportal infusion of MSCs. Coppin and colleagues [50] postulated that intraportal infusion of MSCs in rats leads to transient interruption of the hepatic blood flow as evidenced by intravital microscopy. Wang et al. [51] proved that adipose-derived MSCs transplantation via portal vein improved microcirculation and ameliorated CCl_4_-induced liver fibrosis in rats. Tracing of the transsplenic or rat tail veins transplanted MSCs by liver CT scan showed that only 1% of the transplanted MSCs were able to repopulate in the liver parenchyma in comparison to the transportal route, which provided up to 5-6% repopulation rates [52]. Moreover, recent clinical trials for cell therapy in human liver failure reported the safety and feasibility of surgical intraportal infusion of autologous BM-MSCs for the management of acute liver failure in human patients [53].

The biochemical and histological findings confirmed that liver fibrosis was significantly improved after 60 days of a single intraportal infusion of BM-MSCs, particularly on cessation of CCl_4_ administration. It was obvious that the therapeutic and protective roles of MSCs were abolished with the continuation of the CCl_4_ administration. The MSCs + CCl_4_G showed similar morphometric values to the CCl_4_ group but showed a significant difference with those of MSCs-treated group after withdrawal of CCl_4_.

Regression of fibrosis in the MSCs-transplanted rats was in accordance with the results obtained by Usuneir et al. [54]. He summarized the antifibrotic effects of MSCs as a result of an integrated set of mechanistic actions including (i) immune modulation, (ii) inhibition of TGF*β*-mediated differentiation of various cells into ECM-secreting myofibroblasts by epithelial to mesenchymal transition, (iii) inhibition of oxidative stress, and (iv) matrix remodeling.

The decreased expression for GPC3 simultaneously with the decreased PCNA in the MSCs-transplanted rat livers proved the potential of MSCs in preventing progression of preneoplastic transformation initially triggered by CCl_4_ liver fibrosis. There are controversial theories about the role of MSCs in tumor pathogenesis partly by assuming that they support tumor microenvironments, increase tumor growth, and elicit antitumor immune responses. However, such effects were explained by the probability for existence of a heterogeneous MSCs population originating from contaminated MSCs culture that initially grew slowly and then transformed into cancer cells [55]. Moreover, other research studies reported that using immune-deficient animal models may not be suitable for predicting tumor initiation or progression, because allogeneic MSCs transplantation into immunocompetent mice elicited immune rejection of cells, which prevented the development of tumor in vivo [56].

The major BM-MSCs regenerative potentials have been proposed as twofold mechanisms; one is the improvement of the microenvironments through paracrine effects, and the other is by differentiation for replacement of functional hepatocytes [57]. In particular, after intravenous MSC infusion, paracrine factors released into the blood by circulating MSCs may indirectly influence the fate of distal cells previously compromised by injury or disease. Thus, paracrine factors produced by MSCs do not depend on long-term MSC engraftment nor require the differentiation of MSCs into ectodermal or endodermal tissues [49]. Furthermore, there are several hypotheses that explain the differentiation potential for MSCs. For instance, Dennis et al. [58] suggested that, in MSCs, there are storage genes that can express and adjust differentiation into various lineages when exposed to different conditions.

## 5. Conclusion and Recommendation

The CCl_4_ experimental model proved to be a reproducible model to mimic liver fibrogenesis and microscopic hepatocyte dysplasia. The methods implemented in the present work confirmed that the transportal infusion is an ultimate approach ensuring engraftment of transplanted MSCs. The use of SPIO nanoparticles is a safe material for tracing transplanted MSCs. The applied techniques for morphological, molecular, and biochemical assessment proved the efficiency of transplanted BM/MSCs in restoring the hepatic architecture and functions after the CCl_4_ experimentally induced fibrosis and in regressing the progression of dysplastic changes, particularly after cessation of exposure to the causative agent. Accordingly, it is recommended to advance with research on the positive impact of BM/MSCs in the management of hepatic fibrosis and early dysplasia. The established results on the experimental level should enhance clinical trials that would lead to the application of MSCs as a therapeutic strategy for preventing progression of liver fibrosis and its preneoplastic consequences.

## Figures and Tables

**Table 1:** Comparison between the different studied groups according to ALT, AST (U/ml), total bilirubin (mg/dL), and serum albumin levels (g/dL).

		CG1	CG2	CG3	CG4	LFG	MSCs + CCl_4_G	MSCsG	*p*
ALT (U/ml)	Mean ± SD	18.0 ± 1.7	19.0 ± 2.1	18.3 ± 2.3	17.5 ± 1.2	43.3 ± 4.2^abcd^	35.2 ± 3.9^abcde^	18.2 ± 1.3^ef^	<0.001^*∗*^
AST (U/ml)		31.0 ± 2.5	34.0 ± 4.2	33.7 ± 1.5	32.2 ± 2.4	65.2 ± 4.5^abcd^	54.0 ± 3.1^abcde^	34.5 ± 3.8^ef^	
Total bilirubin (mg/dL)		0.2 ± 0.1	0.3 ± 0.1	0.3 ± 0.1	0.2 ± 0.1	0.8 ± 0.1^abcd^	0.7 ± 0.1^abcd^	0.2 ± 0.1^ef^	
Albumin (g/dL)		3.3 ± 0.3	3.3 ± 0.3	3.1 ± 0.3	3.2 ± 0.2	2.1 ± 0.2^abcd^	2.2 ± 0.2^abcd^	3.1 ± 0.2^ef^	

*p* values for ANOVA test: significance between groups was done using the post hoc test (Tukey). ^a^Significant compared with CG1. ^b^Significant compared with CG2. ^c^Significant compared with CG3. ^d^Significant compared with CG4. ^e^Significant compared with LFG. ^f^Significant compared with MSCs + CCl_4_G, statistically significant at *p* ≤ 0.05.

**Table 2:** Comparison between the different studied groups according to MMP-1 and TIMP-1.

		CG1	CG2	CG3	CG4	LFG	MSCs + CCl_4_G	MSCsG	*p*
MMP-1	Mean ± SD	1.01 ± 0.01	1.05 ± 0.08	1.02 ± 0.04	1.02 ± 0.02	0.41 ± 0.09^abcd^	0.71 ± 0.10^abcde^	1.06 ± 0.08^ef^	<0.001^*∗*^
TIMP-1		0.97 ± 0.05	0.98 ± 0.11	0.99 ± 0.10	0.93 ± 0.09	3.90 ± 0.60^abcd^	2.24 ± 0.57^abcde^	0.91 ± 0.09^ef^	

*p* values for ANOVA test: significance between groups was done using post hoc test (Tukey). ^a^Significant compared with CG1. ^b^Significant compared with CG2. ^c^Significant compared with CG3. ^d^Significant compared with CG4. ^e^Significant compared with LFG. ^f^Significant compared with MSCs + CCl4G, statistically significant at  *p* ≤ 0.05.

## Data Availability

The data used to support the findings of this study are included within the article.
